# Benzalkonium Chloride Suppresses Rabbit Corneal Endothelium Intercellular Gap Junction Communication

**DOI:** 10.1371/journal.pone.0109708

**Published:** 2014-10-09

**Authors:** Zhenhao Zhang, Yue Huang, Hui Xie, Juxin Pan, Fanfei Liu, Xuezhi Li, Wensheng Chen, Jiaoyue Hu, Zuguo Liu

**Affiliations:** 1 Eye Institute and affiliated Xiamen Eye Center of Xiamen University; Fujian Provincial Key Laboratory of Ophthalmology and Vision Science, Xiamen, Fujian, China; 2 Institute of Stem Cell and Regenerative Medicine, Medical College, Xiamen University, Xiamen, Fujian, China; Emory University School of Medicine, United States of America

## Abstract

**Purpose:**

Gap junction intercellular communication (GJIC) plays a critical role in the maintenance of corneal endothelium homeostasis. We determined if benzalkonium chloride (BAK) alters GJIC activity in the rabbit corneal endothelium since it is commonly used as a drug preservative in ocular eyedrop preparations even though it can have cytotoxic effects.

**Methods:**

Thirty-six adult New Zealand albino rabbits were randomly divided into three groups. BAK at 0.01%, 0.05%, and 0.1% was applied twice daily to one eye of each of the rabbits in one of the three groups for seven days. The contralateral untreated eyes were used as controls. Corneal endothelial morphological features were observed by *in vivo* confocal microscopy (IVCM). Immunofluorescent staining resolved changes in gap junction integrity and localization. Western blot analysis and RT-PCR evaluated changes in levels of connexin43 (Cx43) and tight junction zonula occludens-1 (ZO-1) gene and protein expression, respectively. Cx43 and ZO-1 physical interaction was detected by immunoprecipitation (IP). Primary rabbit corneal endothelial cells were cultured in Dulbecco's Modified Eagle Medium (DMEM) containing BAK for 24 hours. The scrape-loading dye transfer technique (SLDT) was used to assess GJIC activity.

**Results:**

Topical administration of BAK (0.05%, 0.1%) dose dependently disrupted corneal endothelial cell morphology, altered Cx43 and ZO-1 distribution and reduced Cx43 expression. BAK also markedly induced increases in Cx43 phosphorylation status concomitant with decreases in the Cx43-ZO-1 protein-protein interaction. These changes were associated with marked declines in GJIC activity.

**Conclusions:**

The dose dependent declines in rabbit corneal endothelial GJIC activity induced by BAK are associated with less Cx43-ZO-1 interaction possibly arising from increases in Cx43 phosphorylation and declines in its protein expression. These novel changes provide additional evidence that BAK containing eyedrop preparations should be used with caution to avoid declines in corneal transparency resulting from losses in GJIC activity and endothelial function.

## Introduction

Corneal endothelial functional activity and homeostasis are essential for this tissue layer to counter the natural tendency of the stroma to imbibe fluid and lose its transparency. [1] The maintenance of corneal deturgescence can be compromised by a variety of stresses that disrupt endothelial integrity. Some of the identified insults include intraocular surgical trauma [2], [3] and corneal transplantation. [4] In addition, ocular surface diseases, topical application of drugs can cause corneal endothelial damage. [5], [6] One reason for this vulnerability is that in humans *in vivo* corneal endothelial cell cannot proliferate since the cells are actively maintained in a non-proliferative, G1-phase–arrested state. [7] Losses in endothelial integrity caused by cell loss are instead compensated for through cell enlargement and stretching at sites proximal to the defect. Given this inability to undergo proliferation, it is essential to characterize specific stress-induced cellular changes since such insight can help design strategies to hasten and improve restoration of transparency caused by an injury. [8]

Cell–cell communication is mediated mainly by gap junctions and is critical for tissue homeostasis. Gap junctions are formed by two hemichannels or connexons which consist of connexins in either homomeric or heteromeric configurations. They are needed for the transfer between cells of small molecules, ions, phosphorylated nucleotides, nutrients, and second messengers (<1KDa) such as cAMP, IP_3_, and Ca^2+^ exchange. [9], [10] Gap junction connectivity is required for numerous cellular processes that underlie the maintenance of tissue homeostasis. They include proliferation, differentiation, and embryonic development. [11] Disruption of gap junction complexes can change cell-cell communication and is associated with certain diseases such as cardio-cerebrovascular disease, [12], [13] tumor, [14], [15] a variety of skin diseases, [16] and congenital cataract. [17] There are approximately 21 human connexin genes and 20 mouse connexin genes which have been identified. [18] Connexin43 (Cx43) is widely expressed and ubiquitously present in a variety of cell types including macrophages. [19] It is also the most common connexin subtype expressed in the corneal endothelium of rat [20], rabbit [21] and human. [22] It has been shown that Cx43 gene knockdown accelerated rabbit corneal epithelial [23] and endothelial [24] wound healing. [25] The fact that losses in gap junctional communication (GJIC) has disparate effects on maintenance of tissue layer homeostasis and wound healing indicates the importance of delineating the effects of specific stresses on GJIC and tissue homeostasis.

Connexins undergo within 2–5 hours rapid turnover involving sequential transitions from synthesis, plaque clustering, junctional formation, final internalization leading to degradation. Cx43 repeatedly cycles through these aforementioned phases. [26]–[28] However, mechanisms that govern the dynamic patterning of gap junctions remain poorly defined. Immunoprecipitation experiments revealed that the carboxyl terminus of Cx43 interacts in other tissues with the second PDZ domain (PDZ2) of Zonula occludens-1 (ZO-1). [29] ZO-1 not only acts as a passive scaffold in organizing gap junction complexes including connexins and cytoskeletals, but also actively participates in the dynamic remodeling of gap junctions, including trafficking of Cx43, gap junction size regulation and Cx43 internalization. [30] Furthermore, gap junction plaques formation and cell communication maintenance are regulated through Cx43 phosphorylation status modulation. [31]

BAK is one of the most commonly used preservatives in ophthalmic preparations. [32] Our previous study demonstrated that BAK could affect changes in ZO-1 distribution and accordingly disrupt corneal endothelial tight junctional barrier function. [33] However, even though it is known that ZO-1 also interacts with the gap junctional component Cx43, the effect of BAK is unknown on corneal endothelial gap junctions. The purpose of this study was to evaluate the effects of BAK on corneal endothelial GJIC activity and on Cx43 gene and protein expression as well as protein-protein interaction between ZO-1 and Cx43.

## Materials and Methods

### Experiment animals

New Zealand albino rabbits (purchased from Shanghai Shilaike Laboratory Animal Center, Shanghai, China) weighing between 1.5 to 2.0 kg were used for this study. Rabbits were kept in a standard room with stable temperature and humidity. All procedures were performed in accordance with ARVO statement for the use of animals in ophthalmic and vision research and approved by the animal ethics committee of Medical College of Xiamen University.

### BAK treatment

According to our previous experimental designs [33] which were slightly modified, thirty-six male adult New Zealand albino rabbits were randomly assigned into three groups. One eye of each rabbit was treated with BAK solution twice daily for seven days. BAK at different concentrations of 0.01%, 0.05%, and 0.1% was applied in different groups, respectively. The contralateral untreated eyes served as controls. At day 7, rabbits were sacrificed with an overdose of pentobarbital sodium, and corneas were removed carefully with ophthalmic surgical scissors.

### Reagents and Antibodies

BAK, dimethyl sulfoxide (DMSO), and Triton X-100, collagenase I and Lucifer Yellow dye were purchased from Sigma Aldrich (St. Louis, MO); Protein A/G PLUS-Agarose Immunoprecipitation Reagent was from Santa Cruz Biotechnology (Santa Cruz, CA); PVDF Western Blotting Membrane was from Roche (Basel, Switzerland); pentobarbital sodium was from Abbott Laboratories (North Chicago, IL); Enhanced chemiluminescence (ECL) kit was obtained from GE Healthcare UK (Chalfont, UK); Mounting medium with 4, 6-diamidino-2-phenylindole (DAPI) and bovine serum albumin (BSA) were from Vector Laboratories (Burlingame CA); Dulbecco's modified Eagle's medium (DMEM) and fetal bovine serum (FBS) were purchased from Life Technologies (Carlsbad, CA); mouse-anti-rabbit ZO-1 antibody, Alexa488-conjugated donkey-anti-mouse IgG, and Alexa555-conjugated donkey-anti-goat IgG were from Life Technologies (Carlsbad, CA); goat polyclonal antibody for Cx43 and P-Cx43, Horseradish peroxidase (HRP)-conjugated donkey anti- goat IgG were from Santa Cruz Biotechnology (Santa Cruz, CA); mouse anti-rabbit β-actin antibody from Sigma Aldrich (St. Louis, MO); Horseradish peroxidase (HRP)-conjugated goat anti- mouse IgG from Merck (Darmstadt, Germany).

### *In Vivo* Confocal Microscopy (IVCM)

After BAK treatment for 7 days, rabbits were anesthetized by intraperitoneal injection of sodium pentobarbital (20 mg/kg; Abbott Laboratories, North Chicago, IL) and intramuscular injection of xylazine (1 mg/kg body weight; Bayer, Shawnee Mission, KS). The Heidelberg Retina Tomograph III/Rostock Cornea Module (Heidelberg Engineer GmbH, Heidelberg, Germany) laser scanning *in vivo* confocal microscopy was used to examine corneal endothelial morphology. Before examination, a drop of carbomer gel (Alcon Laboratories, Fort Worth, TX) was applied to applanating lens. A diode laser was used as a light source at a wavelength of 670-nm. The microscope objective had an immersion lens covered by a polymethyl methacrylate cap (Olympus, Hamburg, Germany). Images consisted of 384×384 pixels, allowing a scanning area of 400 mm^2^ with lateral and vertical resolutions of both 1 mm and a magnification of up to 800 times. The center of the cap was applanated onto the central cornea by adjusting the controller and the central corneal endothelium was examined. More than 10 *in vivo* digital images were captured on the computer. All measurements were performed by a single investigator masked to the specific experimental conditions.

### Immunofluoresecence Staining

Rabbits were sacrificed with an intravenous overdose of pentobarbital sodium, eyes were enucleated and corneas were carefully excised around the limbal rim under a dissecting microscope (Model SZ40; Olympus, Tokyo, Japan). The remaining iris, lens, and retina were thrown away. The freshly isolated corneas were cut into four parts and two quarters were fixed with 4% paraformaldehyde in PBS for 5 minutes at room temperature (RT), followed by acetone for 3 minutes at −20°C. Subsequently, corneas were washed three times in PBS with 1% Triton X-100 and 1% dimethyl sulfoxide (TD buffer) followed by blocking with 2% BSA in PBS for 1 hour at RT. Then corneas were incubated with a polyclonal goat-anti-rabbit Cx43 antibody or mouse-anti-rabbit ZO-1 antibody diluted with 1% BSA overnight at 4°C. After washing three times with TD buffer, corneal tissues were incubated in secondary antibody (donkey-anti-goat IgG conjugated with Alexa Fluor 555 or donkey-anti-mouse IgG conjugated with Alexa Fluor 488) diluted with 1% BSA for 1 hour at RT. After that, tissues were mounted endothelial side up on a slide and stained with DAPI. Omission of primary antibody was used as a negative control. Then corneas were observed under a laser scanning confocal microscope (Olympus Fluoview 1000; Olympus, Japan), and images were captured and processed using Olympus Fluoview software.

### RT-PCR analysis

Corneas were isolated as described above and cut into two parts. Corneal endothelial layer from one part was dissected. Total RNA was extracted with Trizol (Life Technologies, Carlsbad, CA) according to the manufacturer's instructions. 0.5 µg of the RNA were subjected to RT-PCR analysis (First strand cDNA synthesis kit, Fermentas EU) and was performed at 25°C for 5 minutes, 42°C for 2.5 hours, 70°C for 5 minutes, finally cooled to 4°C. After reverse transcription to cDNA, a PCR protocol was implemented to maintain amplification in the exponential phase. Sequence of the PCR primers were designed as follows: Cx43 sense, 5′-GCAAGCTCCTGGACAAAGTC-3′; Cx43 antisense, 5′- CGTTGACACCATCAGTTTGG-3′; ZO-1 sense, 5′-GTCTGCCATTACACGGTCCT-3′; ZO-1 antisense, 5′-GGTCTCTGCTGGCTTGTTTC-3′; Glyceraldehyde-3-phosphate dehydrogenase (GAPDH; internal control) sense, 5′-ACCACAGTCCACGCCATCAC-3′; and GAPDH antisense, 5′-TCCACCACCCTGTTGCTGTA-3′; RT and PCR incubation were performed with a PCR system (GeneAmp 2400-R; Perkin-Elmer, Foster City, CA) and the PCR cycle comprised of incubations at 94°C for 3 minutes, 94°C for 30 seconds, 55°C for 30 seconds, 68°C for 55 seconds, 68°C for 7 minutes. The reaction mixture was finally cooled to 4°C, and the products of amplification were fractionated by electrophoresis on a 2% agarose gel and stained with ethidium bromide (EB). Band intensities were measured by Image Acquisition and Analysis System (UVP, Cambridge, UK). Band intensities were analyzed by image analysis software and those for Cx43 and ZO-1 were normalized by the corresponding value for GAPDH.

### Western blot analysis

Endothelial layers from another part of the corneas was mechanically isolated and washed several times in PBS, then lysed in RIPA buffer containing 50 mM Tris-HCL (PH7.4), 150 mM NaCl, 1%NP-40, 0.5% deoxycholic acid, 0.1%SDS and 1% protease and phosphatase inhibitor cocktail (Thermo Scientific, Rockford, IL). Cell lysates were centrifuged at 15000×g for 25 minutes at 4°C and protein concentration was determined by Bio-Rad DC Protein Assay (Bio-Rad, Hercules, CA). The lysates (equal amounts of total protein) were subjected to SDS-PAGE on 10% (Cx43) and 8% (ZO-1) polyacrylamide gels. Proteins were transferred to PVDF membrane (Millipore) and blocked for 1 hour at RT with TBST (Tris-buffered saline with 0.05% Tween-20) containing 1% BSA. Then membranes were incubated with specific primary antibodies: goat-anti-rabbit Cx43, mouse-anti-rabbit ZO-1 and mouse-anti-rabbit β-actin diluted with blocking buffer (TBST containing 1% BSA) overnight at 4°C. After washing with TBST, membranes were incubated with secondary antibody (HRP conjugated donkey-anti-goat IgG and HRP conjugated goat-anti-mouse IgG) for 1 hour at RT. Immune complexes were detected with an ECL reagent. Band intensities were measured using Molecular Imager ChemiDoc XRS System (Bio-Rad, Hercules, CA) and analyzed with image analysis software.

### Triton X-100 Fraction Isolation

Isolated half of corneal endothelial layer was washed in PBS and lysed in cell lysis buffer containing 1% Triton X-100, 1 mM EDTA and 1% protease and phosphatase inhibitor cocktail. Cell lysates were centrifuged at 15000 g for 25 minutes at 4°C. Supernatant was removed and 1% Triton X-100 insoluble pellet was further lysed in 1×SDS sample buffer by sonication. Both the insoluble and soluble fractions were subjected to western blot analysis using anti-Cx43 antibody separately. Band intensities were measured using Molecular Imager ChemiDoc XRS System and insoluble/soluble Cx43 ratios were calculated.

### Immunoprecipation

Isolated corneal endothelial layers were lysed in cold immunoprecipation buffer (50 mM Tris-HCl (pH 7.5), 150 mM NaCl, 1 mM EDTA, 1% Nonidet P-40, 0.5% sodium deoxycholate, 0.1% SDS, 5 mM NaF, 1 mM Na_3_VO_4_, and 1% protease inhibitor cocktail) and solubilized in cell lysis buffer using a motor-driven tissue homogenate. Cell lysates were centrifuged at 15000 g for 15 minutes at 4°C and protein concentration was determined. Lysates were precleared with magnetic protein G beads (Millipore) for 1 hour at RT to remove nonspecific binding of cell proteins to beads. The beads were discarded and cleared lysates were incubated with goat-anti-rabbit Cx43 antibody and pre-blocked protein A/G agarose beads (Santa Cruz) with BSA overnight at 4°C. After centrifugation at 5000×g for 15minutes, the beads were washed three times in cell lysis buffer and heated at 100°C for 10 minutes in 1×loading buffer. Then proteins to bound beads were subjected to SDS-PAGE analysis using either anti-ZO-1 or anti-Cx43 antibodies as described above.

### Primary Cell Culture and GJIC activity

The scrape-loading dye transfer technique (SLDT) is a method to evaluate GJIC activity by calculating the number of cells containing the dye or measuring the distance of dye permeation through gap junctions. [34] Isolated corneal endothelial layers were digested into single cells in 10 mg/ml collagenase I at 37°C overnight and 0.25% trypsin-EDTA at RT for 2 minutes. Primary rabbit corneal endothelial cells were cultured in DMEM/F12 medium supplemented with 10% FBS and an antibiotic mixture (penicillin 100 U/ml and streptomycin 100 ug/ml) at 37°C in a humidified atmosphere containing 5% CO_2_. BAK (0, 0.00001%, 0.00005% or 0.00025%) was added to the culture medium for 24 hours when cell densities reached about 70% confluence. After the cells were washed with PBS three times, 10 scrapes were made through confluent cells with a sterile pipette tip in the presence of warm PBS containing 1 mg/ml Lucifer Yellow. Cells were further incubated at 37°C and with 5% CO_2_ for 5 minutes. Then Lucifer Yellow was removed, cells were washed with PBS three times and fixed with 4% paraformaldehyde in PBS. The number of cells containing Lucifer Yellow was counted under a laser confocol microscope and 20 sites were measured. GJIC activity was expressed as the mean number of cells containing the dye.

### Statistical Analysis

Quantitative data are presented as mean ± SE and were analyzed with Dunnett's multiple comparison tests. A P value of <0.05 was considered statistically significant.

## Results

### BAK Disrupts Endothelial Integrity

**Figure 1:**
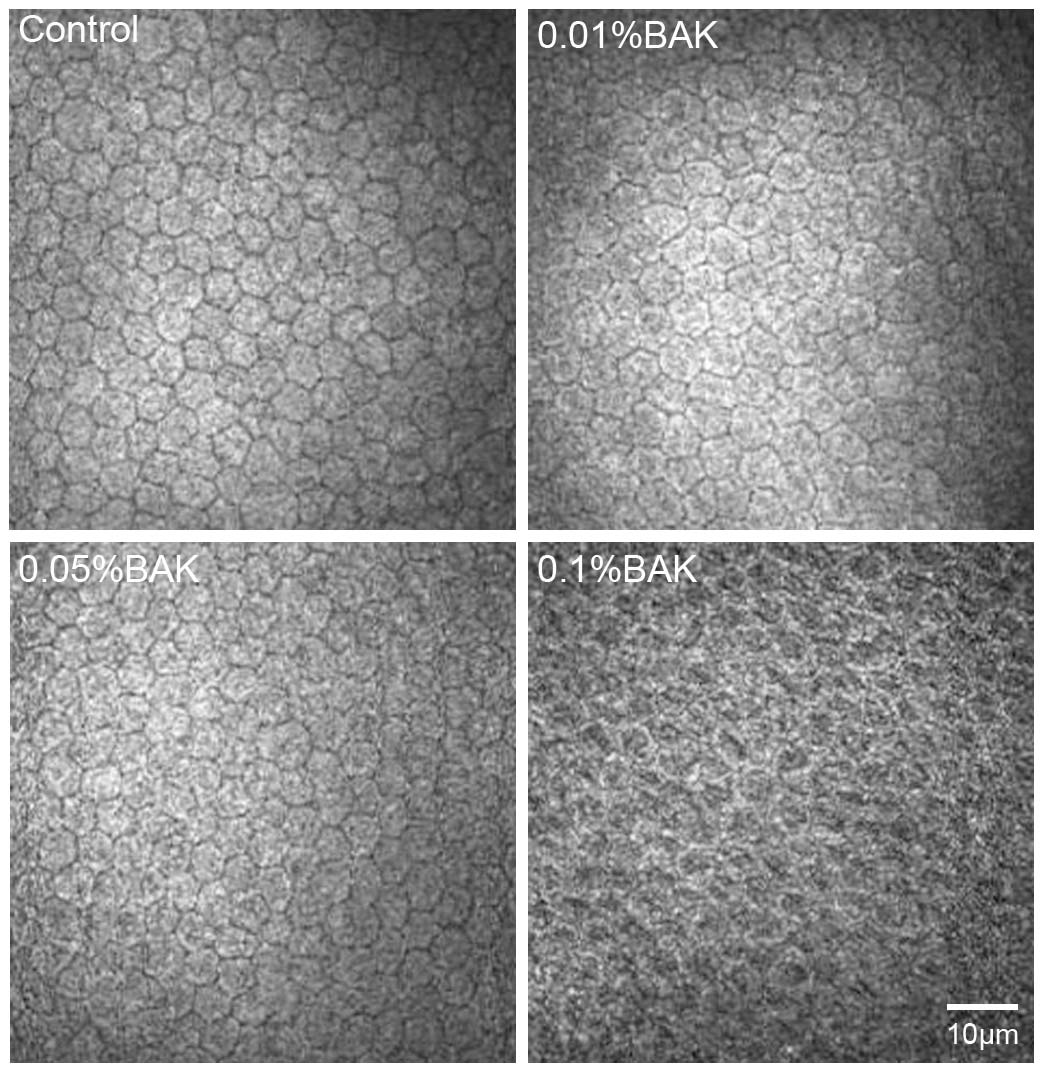
Effects of BAK on rabbit corneal endothelial cell morphology. BAK at 0.01%, 0.05%, and 0.1% was applied twice daily to one eye of each of rabbits for seven days. There was no significant difference of corneal endothelial cells morphology between 0.01% BAK-treated group and control group. In contrast, irregular hexagon cell morphology and blurry boundaries were apparent in 0.05% and 0.1% BAK treated groups. The number of rabbits is nine for three independent experiments (n = 9), three control corneas for IVCM and the remaining control eyes for the culture of corneal endothelial cells.

IVCM images of normal untreated corneal endothelial cells showed that they had hexagonal shapes and were organized in a regular array exhibiting bright cell bodies and dark cell borders. Following topical administration of 0.01% BAK, the cells were undamaged (Fig.1). In contrast, many of cells exposed to either 0.05% or 0.1% BAK lost their hexagonal shape and appeared damaged since their boundaries were distorted and ill defined.

### BAK Treatment Altered Gap Junction Plaques Distribution

**Figure 2:**
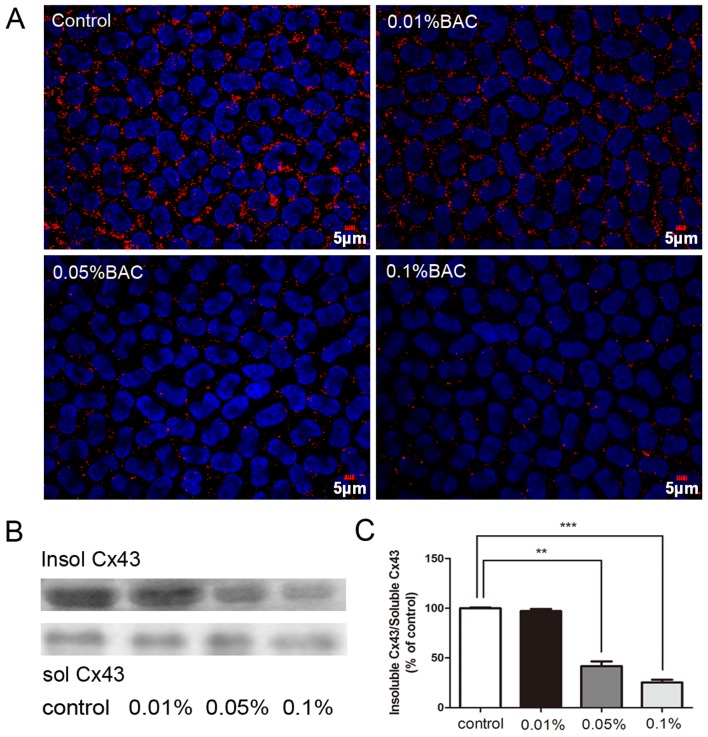
Effects of BAK on Cx43 distribution. In untreated cornea, Cx43 exhibited a great uniformity in appearance with numerous big gap junction plaque. The distribution of Cx43 became disorganized and large gap junction plaques were rarely noticeable in 0.05% and 0.1% BAK-treated group (A). Western blot analysis for 1% Triton X-100 insoluble Cx43 (B) and quantitative analysis of insoluble Cx43 abundance were shown (C). Data are means ± SE from three independent experiments. **P value<0.01, ***P value<0.001 for the indicated comparisons (ANOVA followed by Dunnett's tests).

In untreated corneas, Cx43 was uniformly distributed within the cell plasma membrane in numerous big gap junction plaques. After 0.05% and 0.1% BAK treatment, the readily identifiable plaques along with Cx43 staining became progressively less evident along the endothelial cell borders (Fig. 2A). On the other hand with 0.01% BAK, these changes were less evident than at the two higher BAK concentrations. Associated with the progressive declines in punctate Cx43 staining, western blot analysis shown in Fig. 2B further indicates that gap junctional (1% Triton X-100 -insoluble) Cx43 also fell. Densitometric analysis of this change shown in Fig. 2C indicates that the insoluble/soluble Cx43 ratio reached a value following exposure to 0.1% BAK that was 25.38% of the control value. These declines indicate that BAK treatment led to functional Cx43 gap junction plaque disruption.

### Differential Effects of BAK on Cx43 and ZO-1 Gene and Protein Expression

**Figure 3:**
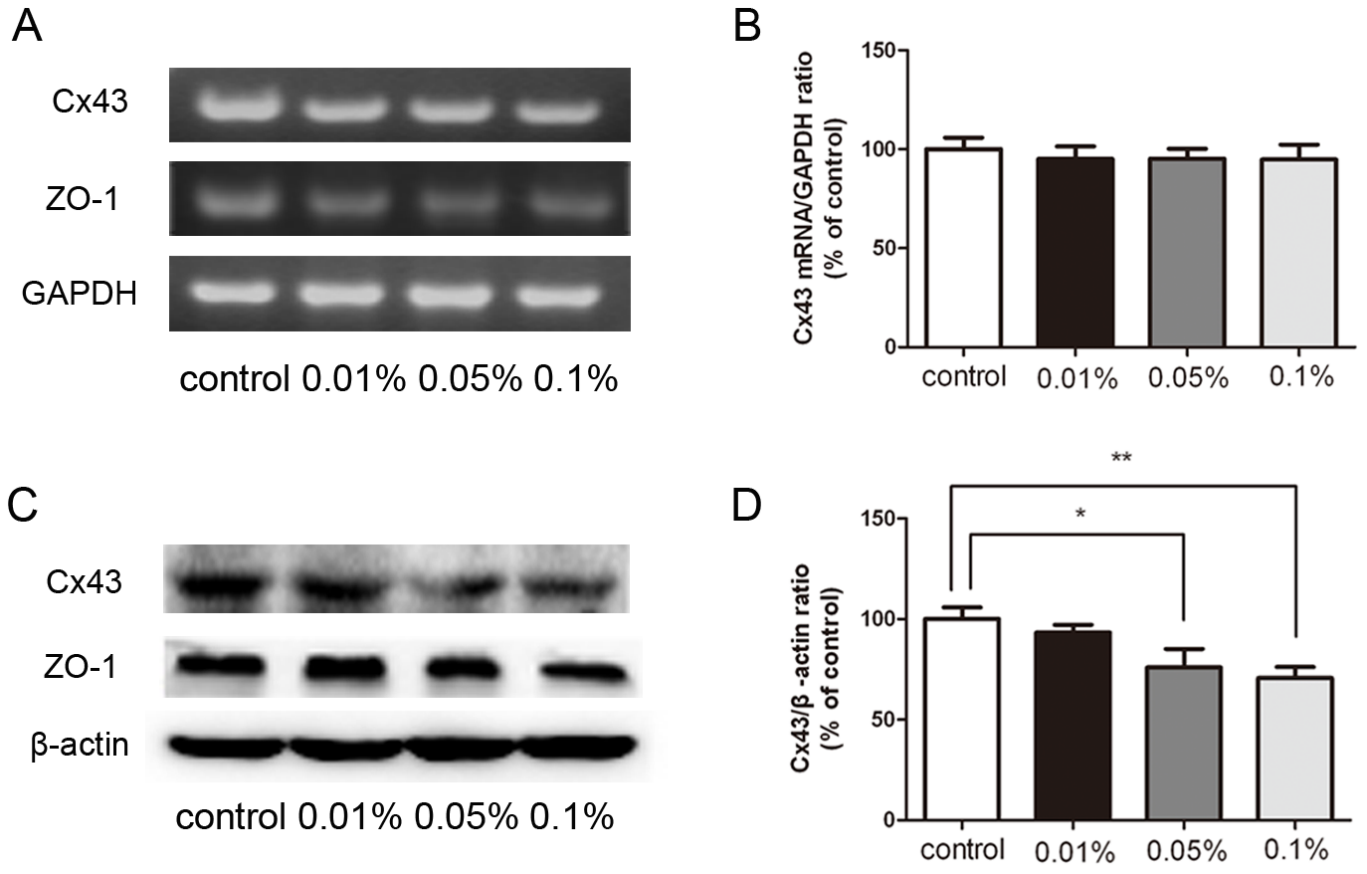
Effect of BAK treatment on Cx43 and ZO-1 expression. The significant decrease of Cx43 protein was induced by topical application of BAK at the concentration of 0.05% and 0.1% (C, D). In comparison, there was no significant decrease of Cx43 and ZO-1 mRNA in BAK treated group (A, B). Similarly, the expression of ZO-1 protein was consistent with mRNA level of ZO-1. Quantitative analysis of Cx43 mRNA and protein in corneal endothelium was shown in (B) and (D) respectively. Data are means ± SE from three independent experiments. *P value<0.05, **P value<0.01 for the indicated comparisons (ANOVA followed by Dunnett's tests).

To determine if the losses in the Triton X-100 insoluble fraction are associated with declines in Cx43 and ZO-1 expression, we performed western blot analysis and RT-PCR, respectively (Fig 3). These approaches indeed revealed that Cx43 protein expression progressively declined during exposure to 0.05% and 0.1% BAK (Fig. 3C, D). On the other hand, RT-PCR analysis showed that in all BAK treated groups Cx43 mRNA expression was essentially invariant (Fig. 3A, B). The abundance of ZO-1 protein and mRNA was also unaffected in BAK treated groups compared with the control group (Fig. 3A and C).

### BAK-Induced Declines in Cx43 and ZO-1 Co-localization and Interaction

**Figure 4:**
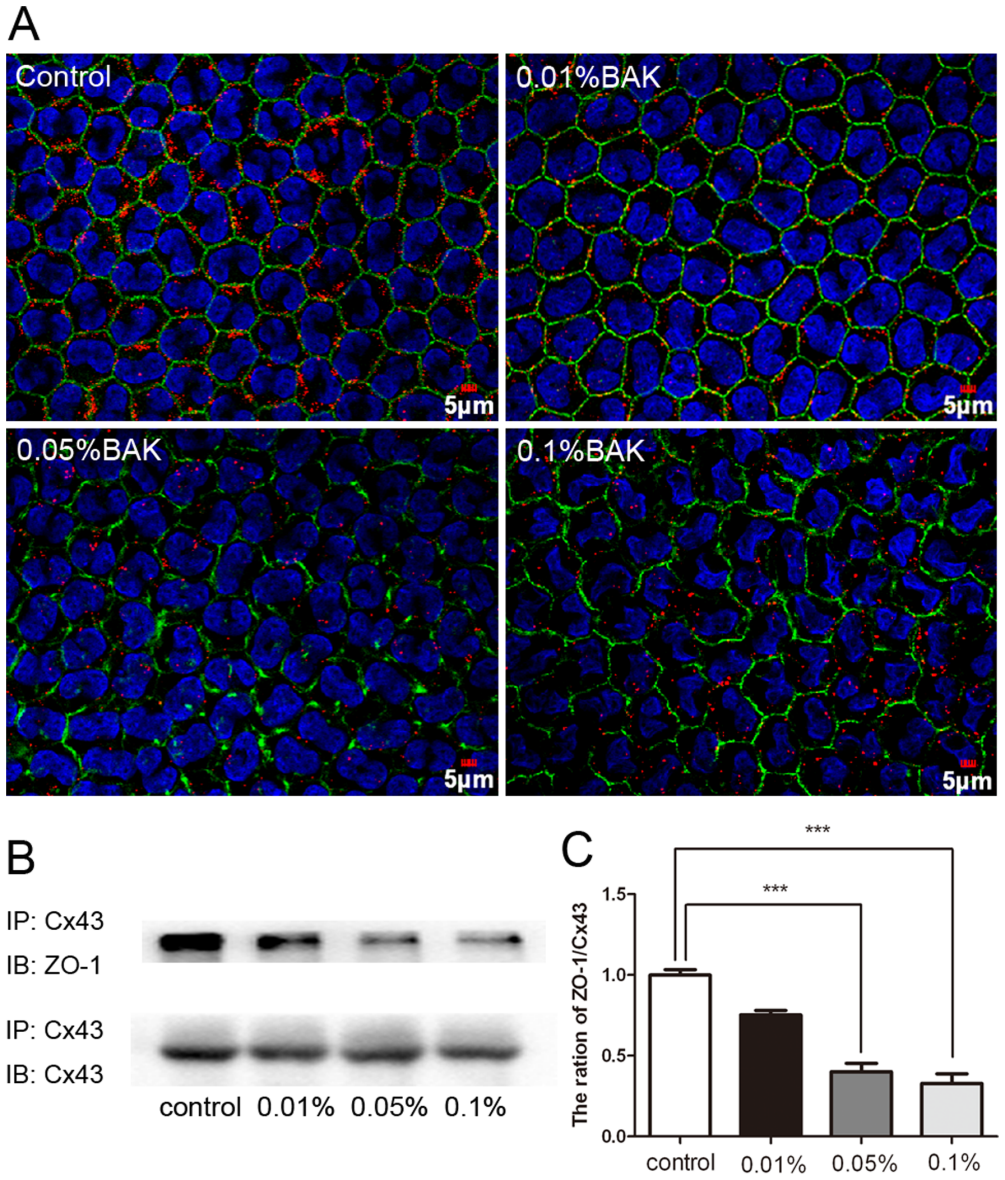
Effect of BAK treatment on co-localization and interaction of Cx43 and ZO-1. Cx43 (red) and ZO-1 (green) co-localization was observed under laser scanning confocal microscope (A). In control group, corneal endothelium presented large gap junction plaques and the co-localization of ZO-1 and Cx43 was obvious. Exposure to 0.05% and 0.1%BAK disturbed the distribution of Cx43 and ZO-1. IP was performed with goat polyclonal anti-Cx43 followed by immunoblotting with mouse monoclonal anti-ZO-1 (B). The ratio of ZO-1 to Cx43 which is normalized with control reduced significantly after BAK treatment especially in 0.05% and 0.1% BAK group (C). Data are means ± SE from three independent experiments. ***P value<0.001 for the indicated comparisons (ANOVA followed by Dunnett's tests).

We previously reported that exposing endothelial cells to BAK dose dependently disrupted their tight junctional barrier properties. ZO-1, a definitive tight junction marker, plays a critical role in maintaining corneal endothelial barrier function. Furthermore, the interactions between Cx43 and ZO-1 determine the gap junction size and GJIC activity. [30] To explore the effects of BAK on Cx43 and ZO-1 association, Cx43 and ZO-1 double immunostaining along with IP were used to assess BAK effects on gap junction plaque integrity. Immunofluorescence staining revealed that in the large gap junction plaques of normal cells, ZO-1 co-localization with Cx43 was very evident. On the other hand, it markedly declined in the 0.05% and 0.1% BAK treated groups (Fig. 4A). These BAK effects agree with the IP result which revealed that the interaction between ZO-1 and Cx43 progressively decreased reaching a value at 0.1% BAK that was only 32.84% of the control group (Fig. 4B, C).

### BAK-Induced Increases in Cx43 Phosphorylation

**Figure 5:**
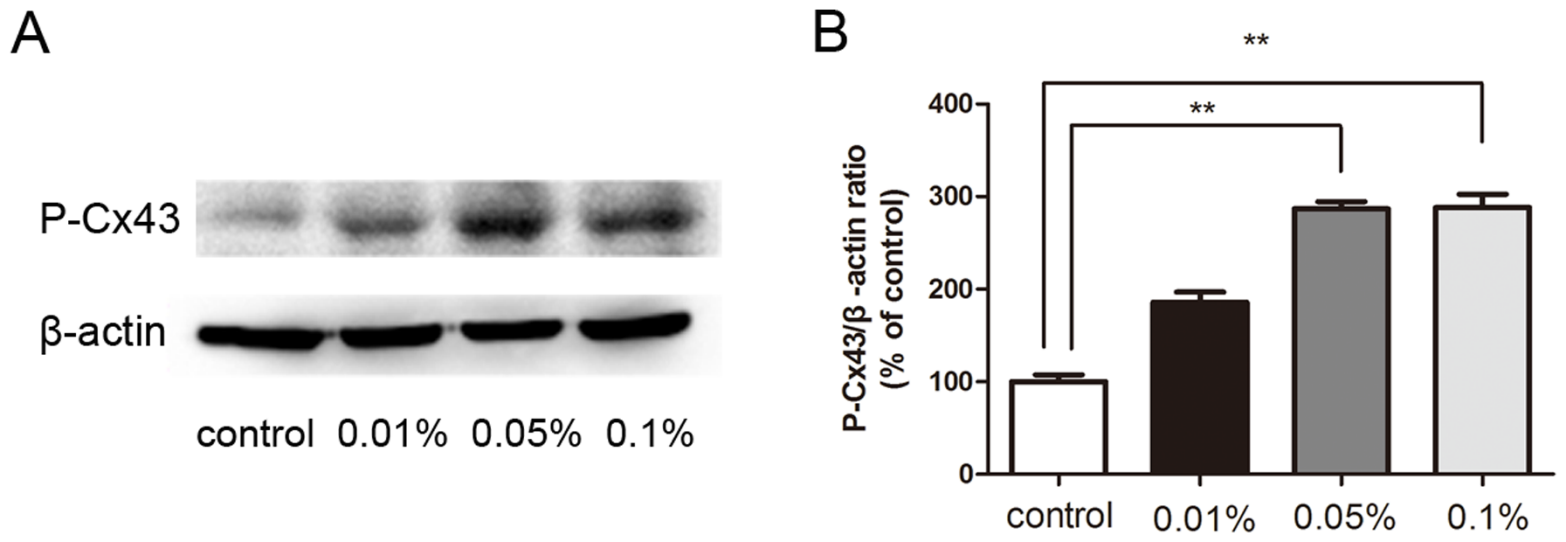
BAK treatment increased Cx43 phosphorylation status. Exposure to BAK induced the increase of phosphorylated Cx43 (A). In 0.05% and 0.1%BAK group, P-Cx43 increased significantly compared with control cornea. Quantitative analysis of P-Cx43 abundance was shown in (B). Data are means ± SE from three independent experiments. **P value<0.01 for the indicated comparisons (ANOVA followed by Dunnett's tests).

Cx43 Phosphorylation status modulation can regulate GJIC activity through a variety of mechanisms by affecting Cx43 plasma membrane transportation, assembly and degradation and modifying gap junctional gating. [35] Significant up-regulation of phosphorylated Cx43 was observed in the 0.05% and 0.1% BAK treated groups, while the 0.01% BAK treated group was not different from the control (Fig. 5A, B). These increases in Cx43 phosphorylation status could contribute to BAK-induced declines in GJIC activity.

### BAK Treatment Suppresses GJIC Activity

**Figure 6:**
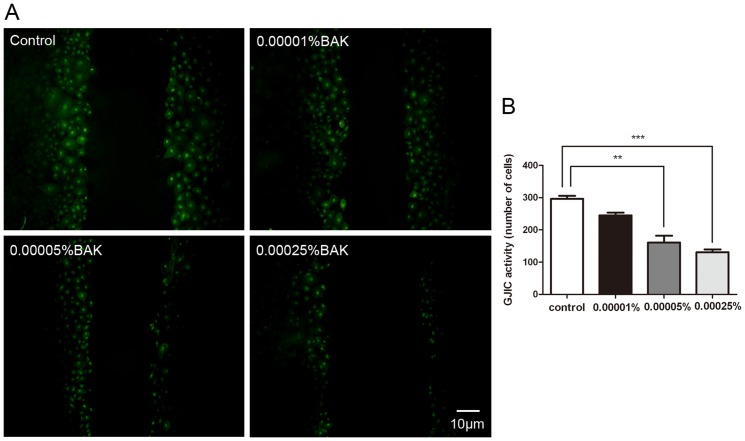
BAK treatment suppressed GJIC activity in primary rabbit corneal endothelial cells. Primary cultured corneal endothelial cells were treated with BAK for 24 hours, BAK was replaced with 1 mg/ml Lucifer Yellow and SLDT assay was performed as described before. Lucifer Yellow transfer through the gap junction was inhibited significantly after 0.00005% and 0.00025% BAK treatment (A). Quantitative analysis of GJIC activity of corneal endothelial cells was shown in (B). Data are means ± SE from three independent experiments. **P value<0.01, ***P value<0.001 for the indicated comparisons (ANOVA followed by Dunnett's tests).

Dye transfer is a commonly used method to evaluate GJIC activity by calculating the number of dye labeled cells or measuring gap junction dye permeation. To determine if the increases in Cx43 phosphorylation status are associated with changes in GJIC activity, we treated the cells for 24 hours with BAK (0, 0.00001%, 0.00005%, and 0.00025%, respectively) and evaluated dye transfer between neighboring cells. These lower BAK concentrations were chosen to minimize cytotoxic effects of prolonged BAK exposure. Gap junctional dye transfer declined at the two higher concentrations (0.00005% and 0.00025%) suggesting an association between increases in Cx43 phosphorylation status and declines in GJIC activity (Fig. 6A, B).

## Discussion

Human corneal endothelial cells cannot replicate and their density declines at an average rate of about 0.6% per year throughout life. [36] Stresses resulting from allograft rejection, inflammation, surgical trauma and topical ophthalmic drug application can induce additional increases in cell loss. As corneal transparency can compromised by BAK containing ophthalmic preparation, there is an extensive literature describing the potential cytotoxic effects on corneal function. We along with others reported that BAK topical application induced ocular surface discomfort, inflammation and disruption of corneal epithelial and endothelial barrier function. [33] In the present study, we examined the effect of BAK on GJIC activity and its physical interaction with a tight junctional barrier protein ZO-1 in rabbit corneal endothelial cells. This was done since it is evident that preservation of GJIC activity and barrier properties is essential to the maintenance of corneal homeostasis. The BAK concentrations are the same that we used in our previous studies in which we described the destructive effects of BAK on rabbit corneal endothelial barrier function. [33]

We show here that topical application of BAK disrupted gap junctional Cx43 distribution (Fig. 2A), down-regulated Cx43 expression (Fig. 3) and suppressed GJIC activity (Fig. 6). Furthermore, these effects were accompanied with increases in Cx43 phosphorylation status (Fig. 5) and concomitant declines in Cx43 interaction with ZO-1 (Fig. 4). These findings suggest that BAK induced disruption of gap junction plaques formation in endothelial cells is associated with declines in Cx43 interaction with ZO-1 and declines in GJIC activity.

Cx43 is an important corneal endothelial gap junctional component which is stably accumulated into gap junctional plaques and its insertion is essential for regulating cell-cell communication under physiological conditions. We found that BAK dose dependently decreased gap junction plaque expression which was associated with declines in Cx43 protein expression and in 1% Triton X-100 extracts the insoluble Cx43 content also fell (Fig.2A–C). These results confirmed the destructive effects of BAK on gap junctions. On the other hand, even after exposure for 7 days to either 0.05% and 0.1% BAK, Cx43 mRNA expression remained unchanged whereas Cx43 protein decreased significantly (Fig.3). This difference suggests that the effects of BAK are restricted to a post-translational modification of Cx43. Many other studies in other tissues concur with this suggestion and indicate that such changes include alteration of Cx43 phosphorylation status and ubiquitination. [37] Declines in Cx43 interaction with ZO-1 are solely due to Cx43 post translational modification since ZO-1 mRNA and protein expression were not affected by BAK treatment in our studies. Recently, *Thomas Tien* et al. found that Cx43 gene silencing with relevant siRNAs decreased ZO-1 and occludin protein expression and increased cell monolayer permeability in rat retinal endothelial cells. [38] It remains to be determined whether Cx43 can regulate ZO-1 expression.

Changes in Cx43 phosphorylation status affect gap junction plaque structure, function and degradation, [35] and regulate GJIC activity. [39], [40] The Cx43 cytoplasmic carboxyl terminal domain is enriched in potential kinase phosphorylation target sites. [41] Gap junction plaques are dynamic cell plasma membrane structures with rapid turnover rates ranging between 2–5 hours. [26]–[28] Regulation of gap junction assembly and turnover controls intercellular communication in all kinds of tissues and cultured cells. [42] Many growth factors, hormones and inflammatory mediators can activate or inhibit intracellular signaling pathways and change connexin phosphorylation status. We found that Cx43 phosphorylation status increased significantly after BAK treatment, which was associated with declines in GJIC activity and Cx43 expression. A variety of kinases have been identified to induce Cx43 phosphorylation including tyrosine protein kinase, mitogen-activated protein (MAP) kinase, protein kinase C (PKC) and casein kinase I. Src is one of the non-receptor tyrosine kinases that can phosphorylate Cx43 carboxyl terminal at Tyr265 site accompanied by suppression of GJIC activity. [41] Further studies are needed to determine the precise signaling pathways that participate in this process.

The cytoplasmic carboxyl terminal domain of Cx43 contains the regulatory binding domains which can modulate Cx43 interactions with other cellular proteins. [41] ZO-1 can bind to the carboxyl terminal intracellular region of Cx43 and is associated with eliciting Cx43 endocytosis. This role for ZO-1 is based on results showing that Hexachlorocyclohexane (HCH), a non-genomic carcinogen that is known to be a potent inducer of Cx43 internalization, decreased the Cx43-ZO-1 association and thus accelerated endocytosis of gap junction plaques. [43] Conversely, *Danny S. Roh* et al. found that even though mitomycin C (MMC) also reduced the interaction of Cx43 and ZO-1, it instead increased the stability of gap junctional plaques in cultured bovine endothelial cells. [25] Our results are consistent with the notion that BAK-induced declines in ZO-1 and Cx43 interaction are associated with gap junctional plaque disruption and declines in GJIC activity. These effects of BAK may be sufficient to account for why at higher concentrations this preservative compromises functional rabbit corneal endothelial cell activity, which is needed to maintain corneal deturgescence and transparency.

After exposing the endothelial cells for 24 hours to Lucifer Yellow, BAK dose dependently reduced GJIC since the number of dye labeled cells declined. There are numerous other ophthalmic mediators that can also reduce GJIC activity. For example, multipurpose solutions used for hydrogel contact lens wear inhibited corneal keratocyte GJIC activity and simultaneously decreased Cx43 abundance. [44] In addition, EGF reduced bovine corneal endothelial cell GJIC activity. [25] So, our experimental results can be readily explained based on the fact that BAK, as a quaternary ammonium cationic surfactant, it disrupted GJIC activity by reducing Cx43expression.

BAK is most often used at a concentration of 0.01% (ranging from 0.004% to 0.025%) in ophthalmic preparations. Its cytotoxic effects are dependent on usage frequency and duration, active ingredients, etc. [32] In this study, we found that BAK is safe to use twice daily for 7 days at a concentration not higher than 0.01%. However, it is still prudent to be on alert for possible declines in endothelial cell GJIC activity if BAK containing ophthalmic preparations are used for longer duration and applied more frequently than twice per day.

In conclusion, topical BAK administration at concentrations above 0.01% induced gap junctional plaque disruption and reduced Cx43 protein expression, but this decline was not attributable to a decrease in Cx43 gene expression. The increases in Cx43phosphorylation status induced by BAK may contribute to observed declines in Cx43-ZO-1 interaction, which in any case are consistent with suppression by this preservative of GJIC activity. These effects of BAK make it even more evident that it is prudent to carefully monitor corneal function when using ophthalmic preparations containing this preservative since cytotoxic effects can severely compromise maintenance of corneal deturgescence and transparency.
